# Course of Lectures for 1856

**Published:** 1858-05

**Authors:** 


### EDITORIAL AND MISCELLANEOUS.

### Course of Lectures for 1858.

Though it is some two weeks previous to the commencement of the course of lectures in the Atlanta Medical College, a number of students have already matriculated, and the prospect for a large class is very flattering. The dome and other improvements upon the College building are rapidly drawing to a close, which will make it one of the most imposing structures of the kind, as well as one of the best adapted to the purpose, in the whole country.

